# Globalization, international law, and emerging infectious diseases.

**DOI:** 10.3201/eid0202.960201

**Published:** 1996

**Authors:** D P Fidler

**Affiliations:** Indiana University School of Law, Bloomington, Indiana 47405-1001, USA. davidfidler@law.indiana.edu

## Abstract

The global nature of the threat posed by new and reemerging infectious diseases will require international cooperation in identifying, controlling, and preventing these diseases. Because of this need for international cooperation, international law will certainly play a role in the global strategy for the control of emerging diseases. Recognizing this fact, the World Health Organization has already proposed revising the International Health Regulations. This article examines some basic problems that the global campaign against emerging infectious diseases might face in applying international law to facilitate international cooperation. The international legal component of the global control strategy for these diseases needs careful attention because of problems inherent in international law, especially as it applies to emerging infections issues.


### Globalization, International Law, and Emerging Infectious Diseases

**David P. Fidler, J.D.**
Indiana University School of Law,
Bloomington, Indiana, USA

The global nature of the threat posed by new and reemerging infectious diseases will require international cooperation in identifying, controlling, and preventing these diseases. Because of this need for international cooperation, international law will certainly play a role in the global strategy for the control of emerging diseases. Recognizing this fact, the World Health Organization has already proposed revising the International Health Regulations. This article examines some basic problems that the global campaign against emerging infectious diseases might face in applying international law to facilitate international cooperation. The international legal component of the global control strategy for these diseases needs careful attention because of problems inherent in international law, especially as it applies to emerging infections issues.

The growing literature on new and reemerging infectious diseases often emphasizes the global nature of their threat; the U.S. Centers for Disease Control and Prevention (CDC) defines these diseases as "diseases of infectious origin whose incidence in humans has increased within the past two decades or threatens to increase in the near future" (1). The World Health Organization has asserted that emerging infections "represent a global threat that will require a coordinated, global response" (2). The threat is global because a disease can emerge anywhere on the planet and spread quickly to other regions through trade and travel. The global challenge of emerging infections has serious consequences for national and international law; a state's ability to deal with them is eroded because microbes do not respect internationally recognized borders (3). Experts grappling with these diseases no longer consider that the pursuit of a strictly national public health policy is adequate. The need for global cooperation increases the importance of international law in the public health arena. Part of the effort to create a global response to emerging infections should be an understanding of the problems that may arise from relying on international law in dealing with these diseases. This article outlines issues that will have to be confronted in using international law to combat emerging infections.

Address for correspondence: David P. Fidler, J.D., Indiana University School of Law, Third Street and Indiana Avenue, Bloomington, IN 47405-1001 USA; fax: 812-855-0555; e-mail: davidfidler@law.indiana.edu.

### Globalization

The assertion that emerging infections are a global problem requiring a global strategy echoes observations made in other spheres of public policy: the traditional distinctions between national and international political, social, and economic activities are losing their importance (4). Globalization is eroding traditional distinctions between domestic and foreign affairs. Globalization has been defined as the "process of denationalization of markets, laws, and politics in the sense of interlacing peoples and individuals for the sake of the common good" (5). Globalization is distinguished from internationalization, which is defined "as a means to enable nation-states to satisfy the national interest in areas where they are incapable of doing so on their own" (5). Internationalization involves cooperation between sovereign states, whereas globalization refers to a process that is undermining or eroding sovereignty.

Globalization arises from the confluence of something old and something new in international relations. It involves the very old process of political and economic intercourse among sovereign states. The new element is the intensification and expansion of such intercourse made possible by technological advances in travel, communications, and computers. Encouraging such intensification and expansion is liberal economic thinking, which posits that economic interdependence makes all states economically better off and builds order and peace in the international system (6).

The changes wrought by new technologies unleashed in the receptive international milieu created by liberal trade and economic policies have led to the belief that these developments are undermining sovereignty. Observers of international relations frequently note that governments no longer have control over economic forces at work within their countries. The speed and volume of international capital flows illustrate the denationalization of economics occurring through the process of globalization (7). Another example is the development of the global company—an enterprise that can no longer be considered national because of the global reach of its operations, financing options, markets, and strategies (7). The globalization of finance and business has ramifications for politics and law as leaders and legal systems adapt to the global era (8).

In public health, a similar combination of old and new factors can be seen. States have historically cooperated on infectious disease control, first through international sanitary treaties and later through the World Health Organization (WHO) (9). While international cooperation is not new, current global circumstances confronting the control of infectious disease are. Globalization is also at work in public health. The assertion that a country cannot tackle emerging infectious diseases by itself demonstrates that public health policy has been denationalized.

Globalization has affected public health in three ways. First, the shrinking of the world by technology and economic interdependence allows diseases to spread globally at rapid speed. Two factors contributing to the global threat from emerging infections stem directly from globalization: the increase in international travel (2, 10) and the increasingly global nature of food handling, processing, and sales (2, 10). HIV/AIDS, tuberculosis, cholera, and malaria represent a few infections that have spread to new regions through global travel and trade (10). The beneficial economic and political consequences of economic interdependence may have negative ramifications for disease control. In the European Union, for example, the free movement of goods, capital, and labor makes it more difficult for member states to protect domestic populations from diseases acquired in other countries (11).

Second, the development of the global market has intensified economic competition and increased pressure on governments to reduce expenditures, including the funding of public health programs, leaving states increasingly unprepared to deal with emerging disease problems. Industrialized as well as developing countries confront deteriorating public health infrastructures (12). Referring to the United States, one author described this deterioration as the "thirdworldization" of the American health care system (13).

Third, public health programs have also "gone global" through WHO and health-related nongovernmental organizations. Medical advances have spread across the planet, improving health worldwide. The worldwide eradication of smallpox in 1977 is a famous example. The global reach of health care advances has, however, a darker side. The globalization of disease control has contributed to the population crisis because people are living longer. Overpopulation creates fertile conditions for the spread of disease: overcrowding, lack of adequate sanitation, and overstretched public health infrastructures (2). Further, the widespread use and misuse of antibiotic treatments has contributed to the development of drug-resistant pathogens (1, 2). Finally, the success of control efforts in previous decades caused interest in infectious diseases to wane in the international medical and scientific communities and is now hampering emerging infectious disease control efforts (14).

### International Solutions to Emerging Infections

International efforts are under way to respond to the threat of emerging infectious diseases. WHO and CDC have drafted action plans that stress the need to strengthen global surveillance of these diseases and to allow the international community to anticipate, recognize, control, and prevent them (1, 14, 15). WHO has also established a new unit to control and prevent emerging infections by mobilizing resources rapidly at the first signs of outbreaks (16). The Pan American Health Organization has also adopted a regional plan for controlling emerging infections in the Americas (17). Health authorities from Central American countries have adopted an emergency plan to control the epidemics of dengue and dengue hemorrhagic fever that recently swept through Central and South America (18). Physicians in the European Union recognize the need for better surveillance of infectious diseases (11). A U.S. government interagency working group has underlined the importance of international cooperation in dealing with the emerging infections threat (19). The U.S. Senate Labor and Human Resources Committee held hearings in October 1995 on "Emerging Infections: A Significant Threat to the Health of the

Nation" (20). At the Halifax Summit in 1995, the major industrialized countries adopted a pilot project called "Toward a Global Health Network" designed to help governments deal with emerging infections and other health problems (19) (Table 1). Clearly, the emerging infections threat and the need for action are on the international diplomatic and public health agendas.

Although international control plans would involve private organizations like universities and nongovernmental organizations, the primary actors on the emerging infections stage are sovereign states. The action plans are predominantly blueprints for cooperation among states and represent a call for the internationalization of responses to a problem caused by globalization. Put another way, the proposed solutions to the emerging infections threat rely on the sovereign state, while the threat feeds off the impotence of the state in addressing global disease problems. When it comes to public health activities, globalization erodes sovereignty, but the proposed solution makes sovereignty and its exercise critical to dealing with the threat of emerging infections.

The consequences of the unavoidable emphasis on international cooperation in the proposed action plans for emerging infections are troubling. To achieve the desired objectives (Table 1), states will have to agree on many issues and translate such agreement into guidelines or rules. International law becomes important to the effort for emerging infections control. Political leaders, diplomats, and scholars have long recognized the weakness of international law in regulating state behavior. At first glance, the prospect of having to rely on a notoriously weak institution of international relations as part of the global effort to combat emerging infections is unsettling.

Table 1. Some common elements of global emerging-disease control plans

| |
|---|
| Strengthen international surveillance networks to detect, control, and reduce emerging diseases. |
| Improve the international public health infrastructure (e.g., laboratories, research facilities, technology, and communications links). |
| Develop better international standards, guidelines, and recommendations. |
| Improve international capabilities to respond to disease outbreaks with adequate medical and scientific resources and expertise. |
| Strengthen international research efforts on emerging diseases, particularly with regards to antibiotic-resistant strains of diseases. |
| Focus attention and resources on training and supporting medical and scientific expertise. |
| Encourage national governments to improve their public health care systems, devote resources to eliminating or controlling causes of emerging diseases and coordinate their public health activities with WHO and the international community. |

Sources: refs. 1, 14, 15, 19.

### International Law and Infectious Disease Control

We might have been less unsettled if our experience with international law in controlling infectious diseases had been more positive. The success of WHO in globalizing disease control programs might suggest that the defects of international law have not hobbled its effectiveness in improving health care worldwide. However, despite having the authority to do so, WHO has been reluctant to use international law (21, 22). The International Health Regulations administered by WHO represent the most important set of international legal rules relating to infectious disease control, but the regulations only apply to plague, yellow fever, and cholera (23). The importance of health is mentioned in international declarations (for example, see the Universal Declaration of Human Rights, art. 25 [1]) and treaties (for example, see the International Covenant on Economic, Social and Cultural Rights, art. 12), leading some legal scholars to argue that international law creates a "right to health" (24); but this "right" does not directly address the control of infectious diseases. WHO has refrained from adopting rules on trade in human blood and organs, which does raise issues of infectious disease control as illustrated by the sale of HIV-contaminated blood in international commerce (25). Issues of disease control also appear in specialized treaty regimes outside WHO, such as treaties controlling marine pollution from ships (26). Other areas of international public health law, for example, rules about infant formula and guidelines on pharmaceutical safety, do not deal with the control of infectious diseases (25).

The effectiveness of existing international law on infectious disease control has been questioned. A 1975 WHO publication stated that the International Health Regulations have not functioned satisfactorily at times of serious disease outbreaks (27). More recently, WHO's efforts with the International Health Regulations have been called a failure, and noncompliance with these regulations has increased in connection with reporting disease outbreaks (25). The HIV/AIDs crisis dramatically illustrated the weaknesses of the health regulations. Since AIDs was not originally (or subsequently) made subject to the regulations, states had, and continue to have, no notification requirements in connection with this new disease. Further, as HIV/AIDs spread globally, many states adopted exclusionary policies that, according to experts, violated provisions of the health regulations (25). In relation to one of the biggest disease crises of this century, parts of the International Health Regulations were irrelevant, and other parts were openly violated.

WHO's reluctance to apply international law has been attributed to its organizational culture, which is dominated by scientists, doctors, and medical experts. Perhaps the current weakness of international law on infectious disease control reflects WHO's nonlegal strategy rather than the inherent problems in international law itself. In connection with emerging infections, however, WHO is advocating an international legal strategy by recommending revision of the International Health Regulations (28). This recommendation suggests that WHO acknowledges the need for international legal agreement in dealing with emerging infections. The global threat posed by these infections represents in many ways a test case for international public health law.

### The Challenge to International Law

The threat of emerging infectious diseases poses two challenges to international law: first, the emerging infections problem exacerbates basic weaknesses in the law. Second, these infections pose specific difficulties in the law, which are related to the nature of disease and its prevention.

### Basic Weaknesses

The effectiveness of international law depends on the consent of states, which means that sovereignty and its exercise determine the fate of international legal rules (29). In adopting a legal strategy for its emerging infectious disease action plan, WHO has to convince its member states to take certain actions in response to disease emergence. The sovereignty of states looms large in formulating a global response to emerging infections, despite the fact that the process of globalization undermines the sovereignty of the state to deal nationally with these infections. In other words, the problem by-passes the state, but the solution has to rely on the state through the medium of international law. The central importance of the state and its sovereignty constitutes a basic weakness in international law because international legal rules tend to reflect the compromises necessary to achieve agreement and the unwillingness of states to restrict their freedom of action through international law. Part of the reason that the existing International Health Regulations cover only a few diseases might be the unwillingness of WHO member states to commit to more serious infectious disease control measures. The vagueness and lack of specificity in the so-called "right to health" also illustrate this problem. What is scientifically and medically necessary to combat emerging diseases may not be what states are willing to agree to undertake.

A second basic weakness follows from the "sovereignty problem"—the lack of effective enforcement of international law. States often agree to an international legal obligation without any serious intent of fulfilling it. The alleged failure of the International Health Regulations may be due to the failure of WHO member states to fulfill the duties they accepted. Neither the regulations nor WHO has any power to enforce compliance (25). An international legal regime on emerging diseases would also face this enforcement problem.

### Specific Difficulties

The very nature of the emerging disease threat poses special difficulties for international law. The global scope of the problem necessitates agreement by most states to control emerging diseases. If any major country or group of countries does not participate, a gap in the global surveillance and control network threatens the efficacy of the entire effort. The negotiation of agreements involving many states is usually difficult, because each state knows that its nonparticipation threatens the success of the entire venture. This problem has occurred in international environmental law, where global regimes have been needed to deal adequately with environmental threats, such as ozone depletion.

A second specific difficulty arises from the extent of medical and scientific resources needed to establish an effective global surveillance and control network for emerging diseases. Fundamental aspects of the proposed action plans involve improving surveillance networks, public health infrastructures, scientific research, and medical and scientific training (Table 1). Some states, particularly in the developing world, do not have the medical, scientific, and financial resources to undertake such measures. Unless more affluent countries provide the resources, developing states may use the inequity of wealth in the international system as an argument to complicate negotiating a global agreement. The so-called "North-South problem" has made the negotiation of international environmental agreements more difficult, as developing countries have bargained for more lenient treatment or a transfer of resources from affluent countries to help them improve environmental protection. A similar dynamic may appear in any negotiations for a global emerging disease effort. The U.S. interagency working group on emerging diseases has observed that major U.S. contributions to developing countries for emerging disease control purposes "is not a likely prospect during this period of deficit reduction and downsizing" (19), which suggests that resource availability will probably complicate international efforts in this area.

The problems associated with using international law in a global strategy to combat emerging diseases raise the question whether international law can provide an adequate foundation for the control of these diseases. The uncomfortable position of having no choice but to rely on international law when its weaknesses are substantial highlights the importance of thinking through the international legal aspects of a global emerging disease plan carefully.

### WHO's Proposed Legal Strategy

WHO wants to revise the International Health Regulations as part of its global emerging disease strategy (28). WHO's proposal deserves some critical attention. It is not clear that the organization has adequate authority to incorporate comprehensive emerging disease control measures within the international regulations. Under Article 21 of the WHO Constitution, the World Health Assembly can adopt binding regulations in sanitary and quarantine requirements and other procedures to prevent the international spread of disease (22). The World Health Assembly adopted the International Health Regulations under Article 21. While Article 21 and the regulations are relevant to emerging disease control efforts, it is doubtful whether the regulations can serve as a foundation for a comprehensive emerging disease control plan. The disease-outbreak notification requirements in the regulations could be expanded to include more diseases, but nothing in Article 21 gives the World Health Assembly the authority to require WHO member states to strengthen public health infrastructures, which is considered critical in the emerging disease actions plans proposed to date (Table 1). It has been argued that attempting to address such infrastructure problems "is a solution which cannot be obtained by an international instrument but only by the improvement of the health conditions of the peoples of WHO's member states" (30). But, as the history of administering the International Health Regulations has shown, notification requirements have not worked satisfactorily and are weakened by the absence of adequate public health resources. Further, Article 22 of the WHO Constitution makes regulations promulgated under Article 21 automatically binding on WHO member states, except for member states that reject such regulations or make reservations thereto (31). Article 22 relates to the sovereignty problem and may deter WHO member states from agreeing to serious revisions of the regulations. Analysis of the regulations may question the wisdom of using the regulations as the legal basis for dealing with emerging diseases.

The World Health Assembly has the power to adopt conventions or agreements within WHO's competence (21). The Assembly could use this authority to address aspects of the global emerging disease control strategy that cannot be handled with a revision of the regulations. However, parceling up emerging disease control measures between the International Health Regulations and separate agreements would be legally complicated. Further, WHO has not used this power to adopt conventions or agreements, which explains its unwillingness to explore all legal options open to it.

### Possible Alternative Legal Strategies

Alternative legal strategies to revising the International Health Regulations range from reliance on the development of customary international law to the adoption of multilateral treaties specifically on emerging-disease control (Table 2). An issue related to these alternative approaches is the substantive nature of the obligations contained in legal documents. We have to ask not only how states might agree on control rules but also what these states might agree to do. The proposed revision of the regulations

Table 2. Alternative international legal strategies to revising the International Health Regulations

| Alternative legal strategies | Possible advantages | Possible disadvantages |
|---|---|---|
| 1. WHA incorporates emerging disease control as part of the proposed World Health Charter scheduled for initial negotiations in 1997 | Integrates emerging disease control measures into the overall WHO approach to international health issues | a. Emerging disease control would not be primary focus<br>b. World Health Charter is likely to be more aspirational than obligatory |
| 2. WHA adopts an emerging disease–specific convention under Article 19 of the WHO Constitution | a. Avoids IHR model<br>b. Has potential to set out comprehensive global approach to emerging diseases | a. WHA has no experience with using Article 19<br>b. Large multinational treaties tend to contain general obligations rather than specific duties |
| 3. States negotiate a framework multilateral treaty on general emerging disease obligations, accompanied by disease-specific or region-specific protocols containing detailed and specific commitments on emerging disease control | a. Takes emerging disease control out of WHO, eliminating problem of WHO's reluctance to use international law<br>b. Allows for new protocols to be adopted for new diseases<br>c. Framework-protocol approach has been used with some success in international environmental law on ozone depletion | a. WHO has to play central role in any emerging disease plan<br>b. Framework-protocol approach might not be appropriate model for emerging disease control because the emerging disease problem differs from ozone depletion |
| 4. Encourage regional arrangements and integrate them into global regime over time | a. Builds on strong regional systems of cooperation and coordination<br>b. Offers "legal laboratories" to try various approaches to emerging disease control<br>c. Avoids diplomatic headaches involved in trying to negotiate truly global legal regimes | a. Emerging diseases require a global approach not just a regional approach<br>b. Amounts to emerging disease control for rich regions, leaving many developing countries outside legal regime<br>c. Risks inconsistencies in how emerging diseases are handled by different regions |
| 5. Encourage a bilateral approach in which individual countries negotiate detailed and specific commitments on emerging diseases and perhaps condition trade benefits and aid on emerging disease performance | a. Gives states flexibility in constructing legal obligations<br>b. Permits possibility for sanctions for failure to live up to emerging disease obligations | a. Does not address global nature of emerging disease problem<br>b. Sanctions element is unrealistic and might be unfair to developing countries lacking the resources necessary to implement adequate emerging disease control measures |
| 6. Incorporate emerging disease control as part of international "right to health," making emerging diseases a human rights issue | a. Links emerging disease control with larger, powerful concepts of human welfare<br>b. Builds on existing international law on the "right to health" | a. International "right to health" has no definitive meaning or scope and thus is a bad foundation for emerging disease control<br>b. Human rights are inherently divisive in the international system; linkage with such a controversial area would hurt emerging disease control prospects |
| 7. Rely on customary international law to develop emerging disease-control norms | Customary international norms on emerging disease control would be binding on all states except persistent objectors | a. It will be nearly impossible to develop general and uniform state practice recognized by states as legally binding in the emerging disease-control area<br>b. Any customary norms that might form will probably be vague and hard to identify definitively<br>c. Customary norms can take a very long time to develop |

WHA = World Health Assembly; WHO = World Health Organization; IHR = International Health Regulations.

apparently would only apply the notification duties (currently found in the regulations) to more diseases. As indicated earlier, WHO cannot address in its revision of the regulations any of the improvements in public health infrastructures, surveillance networks, scientific research, or medical and scientific training at the heart of proposed emerging disease action plans. Further, it is not clear whether WHO intends to supplement expanded notification duties with any mechanism to monitor or enforce such duties.

International environmental law had to overcome some of the same obstacles encountered by WHO's international legal effort for emerging disease control. States realized that they could not handle global environmental problems without international cooperation and rules (32). Further, states knew that addressing environmental concerns would require changes for governments and companies within states and that developing states might have financial and technological difficulties implementing international agreements (32). In developing international environmental law, states, international organizations, and nongovernmental organizations did not rely on old approaches but instead crafted new international legal rules to deal with the global nature of the threats posed, the resource issue, and compliance and enforcement problems (33). Whether international environmental law has been successful is controversial; but it is important that states have not been willing to admit that improving environmental conditions within states is a solution that cannot be obtained by international agreements. Models and precedents from international environmental law are not in all respects helpful to the challenge of emerging-disease control; but, at the very least, those grappling with an international strategy for the emerging-disease threat could analyze international environmental law and other innovative legal responses to globalization to look for ways of making WHO's international legal strategy on emerging diseases as effective as possible.

Those currently designing global emerging-disease control strategies will eventually have to translate what is scientifically and medically needed to combat these diseases into international agreement and cooperation through international law. The movement from science and medicine into the realm of international law will not be easy. Relying on the International Health Regulations as the centerpiece of international law on emerging-disease control may not be the most effective international legal strategy. Whatever international legal approach is eventually taken will have to confront somehow a fundamental paradox: globalization jeopardizes disease control nationally by eroding sovereignty, while the need for international solutions allows sovereignty to frustrate disease control internationally. The combination of the process of globalization and the unavoidable need to rely on international law produces a most unattractive medium in which to wage potentially one of the most important medical and scientific endeavors in history.

*Dr. Fidler is associate professor of law at Indiana University School of Law, Bloomington, where he teaches public and private international law. He has a master of philosophy (M.Phil.) degree in international relations from Oxford University, a J.D. from Harvard Law School, and a bachelor of civil law degree from Oxford University. Before joining the faculty at Indiana University School of Law, Mr. Fidler practiced law with Sullivan & Cromwell in London, England, and with Stinson, Mag & Fizzell, P.C., in Kansas City, Missouri.*

### Acknowledgments

I thank Jack Bobo, research assistant, for his valuable work during the preparation of this article, and Lane Porter and Allyn Lise Taylor for their helpful comments on earlier drafts of this article. I also thank my colleagues on the faculty of Indiana University School of Law for their helpful comments on an earlier draft presented at a faculty colloquium.

### References

1. Centers for Disease Control and Prevention. Addressing emerging infectious disease threats: a prevention strategy for the United States. Atlanta: U.S. Department of Health and Human Services, Public Health Service, 1994.
2. World Health Organization. Communicable disease prevention and control: new, emerging, and re-emerging infectious diseases. WHO Doc. A48/15; Feb. 22, 1995.
3. Garrett L. The return of infectious disease. Foreign Affairs Jan.-Feb. 1996;66-79.
4. Aman AC. Introduction. Indiana Journal of Global Legal Studies 1993;I:1-8.
5. Delbrück J. Globalization of law, politics, and markets—implications for domestic law—a European perspective. Indiana Journal of Global Legal Studies 1993;I:9-36.

6. Gilpin R. The political economy of international relations. Princeton, NJ: Princeton University Press, 1987.
7. Kennedy P. Preparing for the twenty-first century. London: Harper Collins, 1993.
8. Shapiro M. The globalization of law. Indiana Journal of Global Legal Studies 1993;I:37-64.
9. McNeill WH. Plagues and peoples. New York: Doubleday, 1976.
10. Wilson ME. Travel and the emergence of infectious diseases. Emerging Infectious Diseases 1995;1:39-46.
11. Desenclos J-C, Bijkerk H, Husiman J. Variations in national infectious disease surveillance in Europe. Lancet 1993;341:1003-6.
12. Berkelman RL, Bryan RT, Osterholm MT, Leduc JW, Hughes JM. Infectious disease surveillance: a crumbling foundation. Science 1994;264:368-70.
13. Garrett L. The coming plague: newly emerging diseases in a world out of balance. New York: Penguin Books, 1994.
14. Emerging infectious diseases: memorandum from a WHO meeting. Bull World Health Organ 1994;72:845-50.
15. World Health Assembly. Communicable diseases prevention and control: new, emerging, and re-emerging infectious diseases. WHO Doc. WHA 48.13, May 12, 1995.
16. World Health Organization. Press Release. WHO/75, Oct. 17, 1995.
17. Epstein DB. Recommendations for a regional strategy for the prevention and control of emerging infectious diseases in the Americas. Emerging Infectious Diseases 1995;1:103-5.
18. World Health Organization. Press Release. WHO/72, Sept. 28, 1995.
19. National Science and Technology Council Committee on International Science, Engineering, and Technology Working Group on Emerging and Re-Emerging Infectious Diseases. Infectious disease—a global health threat. Washington, DC: CISET, 1995.
20. Emerging infections: a significant threat to the nation's health: hearing before the comm. on labor and human resources. 104th Cong., 1st Sess. 298 (1995).
21. WHO Constitution, art. 19.
22. WHO Constitution, art. 21.
23. World Health Organization. International health regulations. Geneva: World Health Organization, 1983.
24. Taylor AL. Making the world health organization work: a legal framework for universal access to conditions for health. Am J Law Med 1992;18:301-46.
25. Tomasevski K. Health. In: Scachter O, Joyner CC, editors. United Nations Legal Order. Cambridge, UK: Cambridge University Press, 1995.
26. Churchill RR, Lowe AV. The law of the sea. 2nd ed. Manchester, UK: Manchester University Press, 1988.
27. Delon PJ. The International Health Regulations: a practical guide. Geneva: World Health Organization, 1975.
28. World Health Assembly. Revision and Updating of the International Health Regulations. WHO Doc. WHA 48.7, May 12, 1995.
29. Brownlie I. Principles of public international law. 4th ed. Oxford, UK: Oxford University Press, 1990.
30. Fluss S. International public health law: an overview. In: Oxford Textbook of Public Health. In press.
31. WHO Constitution, art. 22.
32. Hurrell A, Kingsbury B. The international politics of the environment: an introduction. In: Hurrell A, Kingsbury B, editors. The international politics of the environment. Oxford, UK: Oxford University Press, 1992.
33. Birnie P. International environmental law: its adequacy for present and future needs. In: Hurrell A, Kingsbury B, editors. The international politics of the environment. Oxford, UK: Oxford University Press, 1992.
